# Overexpression of miR390b promotes stem elongation and height growth in *Populus*

**DOI:** 10.1093/hr/uhac258

**Published:** 2022-11-21

**Authors:** Qiaofang Shi, Dongdong Tian, Jieyu Wang, Aoli Chen, Yuqing Miao, Yiming Chen, Jun Li, Xiaomeng Wu, Bo Zheng, Wenwu Guo, Xueping Shi

**Affiliations:** Key Laboratory of Horticultural Plant Biology of Ministry of Education, College of Horticulture and Forestry Sciences, Huazhong Agricultural University, Wuhan 430070, China; Poplar Research Center, Huazhong Agricultural University, Wuhan 430070, China; Key Laboratory of Horticultural Plant Biology of Ministry of Education, College of Horticulture and Forestry Sciences, Huazhong Agricultural University, Wuhan 430070, China; Tobacco Research Institute, Chinese Academy of Agricultural Sciences, Qingdao 266101, China; Key Laboratory of Horticultural Plant Biology of Ministry of Education, College of Horticulture and Forestry Sciences, Huazhong Agricultural University, Wuhan 430070, China; Poplar Research Center, Huazhong Agricultural University, Wuhan 430070, China; Key Laboratory of Horticultural Plant Biology of Ministry of Education, College of Horticulture and Forestry Sciences, Huazhong Agricultural University, Wuhan 430070, China; Key Laboratory of Horticultural Plant Biology of Ministry of Education, College of Horticulture and Forestry Sciences, Huazhong Agricultural University, Wuhan 430070, China; Key Laboratory of Horticultural Plant Biology of Ministry of Education, College of Horticulture and Forestry Sciences, Huazhong Agricultural University, Wuhan 430070, China; Poplar Research Center, Huazhong Agricultural University, Wuhan 430070, China; Key Laboratory of Horticultural Plant Biology of Ministry of Education, College of Horticulture and Forestry Sciences, Huazhong Agricultural University, Wuhan 430070, China; Poplar Research Center, Huazhong Agricultural University, Wuhan 430070, China; Key Laboratory of Horticultural Plant Biology of Ministry of Education, College of Horticulture and Forestry Sciences, Huazhong Agricultural University, Wuhan 430070, China; Key Laboratory of Horticultural Plant Biology of Ministry of Education, College of Horticulture and Forestry Sciences, Huazhong Agricultural University, Wuhan 430070, China; Poplar Research Center, Huazhong Agricultural University, Wuhan 430070, China; Key Laboratory of Horticultural Plant Biology of Ministry of Education, College of Horticulture and Forestry Sciences, Huazhong Agricultural University, Wuhan 430070, China; Key Laboratory of Horticultural Plant Biology of Ministry of Education, College of Horticulture and Forestry Sciences, Huazhong Agricultural University, Wuhan 430070, China; Poplar Research Center, Huazhong Agricultural University, Wuhan 430070, China

## Abstract

MicroRNA390 (miR390) is involved in plant growth and development by down-regulating the expression of the downstream genes *trans-acting short interfering RNA3* (*TAS3*) and *AUXIN RESPONSE FACTORs* (*ARFs*). There is a scarcity of research on the involvement of the miR390-*TAS3-ARFs* pathway in the stem development of *Populus*. Here, differentially expressed miRNAs during poplar stem development were screened by small RNA sequencing analysis, and a novel function of miR390b in stem development was revealed. Overexpression of miR390b (OE-miR390b) resulted in a large increase in the number of xylem fiber cells and a slight decrease in the cell length at the longitudinal axis. Overall increases in stem elongation and plant height were observed in the OE-miR390b plants. According to transcriptome sequencing results and transient co-expression analysis, *TAS3.1* and *TAS3.2* were identified as the target genes of miR390 in poplar and were negatively regulated by miR390 in the apex. The transcription levels of *ARF3.2* and *ARF4* were significantly repressed in OE-miR390b plants and strongly negatively correlated with the number of xylem fiber cells along the longitudinal axis. These findings indicate that the conserved miR390-*TAS3*-*ARFs* pathway in poplar is involved in stem elongation and plant height growth.

## Introduction

*Populus* is a model plant for studying the growth and development of trees and perennial woody plants. Meanwhile, *Populus* is a significant afforestation species and a major raw material for various timber industries. Secondary growth of woody plants is the basis for the wood formation and its prerequisites are primary growth and stem elongation [1]. Elongation growth usually occurs during the transition from primary to secondary growth of stem, and the associated molecular regulatory mechanisms are critical for improving wood quality and yield through genetic engineering. Some studies have been conducted to validate the regulatory functions of genes related to stem elongation and plant height in poplar. For example, overexpression of the heterologous *PHYTOCHROME A* (*PHYA*) was found to result in the accumulation of *CENTRORADIALIS-LIKE1* (*CENL1*) in the rib meristem under short-day treatment, thereby promoting stem elongation and affecting plant height [2]. Overexpression of the cytokinin metabolism gene *CYTOKININ OXIDASE* (*CKX*) led to a decrease in cytokinin levels, which further significantly inhibited stem elongation [3]. Expression of both *GA20-oxidase*, a key gene of the gibberellin synthesis pathway, and *GID1*, a GA receptor gene, positively regulated internode elongation and plant height growth [4, 5]. Recently, two tandem CCCH zinc finger genes, *C3H17* and *C3H18*, targeted by *MYB3* and *MYB21*, were reported to affect stem elongation and plant height [6, 7]. However, the molecular mechanisms regulating stem elongation and plant height in poplar remain unclear.

As key regulatory factors in plant growth and development, microRNAs (miRNAs) are a class of small single-stranded non-coding RNAs (~21 nucleotides) that are involved in the negative regulation of target gene expression primarily by inhibiting the initiation of mRNA translation of the target gene or by directly cleaving the mRNA of the target gene [8]. Numerous miRNAs were demonstrated to serve as important regulators of wood formation. Specifically, the functional inhibition of both miR393 and miR6443 increased the lignin content, and the overexpression of both miR397 and miR6443 decreased the lignin content, indicating that miR393, miR397, and miR6443 are negative regulators of lignin biosynthesis in poplar [9–11]. The gene *TEOSINTE BRANCHED 1/CYCLOIDEA/PCF 20* (*TCP20*), which is negatively regulated by miR319, promotes vascular formation layer proliferation through interaction with *WUSCHEL-RELATED HOMEOBOX 4* (*WOX4*) and activates *WND6* transcription to promote secondary xylem differentiation [12]. By regulating the target class III homeodomain-leucine zipper (HD-Zip III), miR166 participates in poplar secondary growth and the initiation of cambium [13, 14]. Moreover, various omics data have been continuously reported in terms of stem development in different poplar varieties [15–18]. However, there is a scarcity of research on miRNA in poplar stem elongation.

In plants, miR390 cleaves the transcript of the target gene *trans-acting siRNA 3* (*TAS3*) to produce *trans-acting small interfering RNAs* (*tasiRNAs*), also known as *tasiARFs*, which can cleave transcripts of *AUXIN RESPONSE FACTOR* (*ARF*) *2*, *ARF3*, and *ARF4* [19, 20]. The regulatory functions of miR390/*TAS3*/*ARFs* have been demonstrated in several species, such as the involvement in lateral root (LR) formation, floral meristem determinacy, and seed size in *Arabidopsis* [21–24], somatic embryogenesis of longan [25], the establishment of leaf polarity in maize [26], development phase transition of *Physcomitrella patens* [27], root nodule symbiosis of *Lotus japonicus* [28], nodulation formation of *Medicago truncatula* [29], maintenance of normal development of vascular tissue and shoot apical meristem (SAM) in rice [30], and response to biotic stress [31] and abiotic stress [32]. In *Populus*, the expression level of miR390 was positively correlated with vascular cambial activity [16]. The functions of miR390 in response to salt stress promoting LR development has been recently verified in *Populus* [33]. However, there is limited research on the involvement of miR390/*TAS3*/*ARFs* in plant stem elongation. In the present study, differentially expressed miR390 was mined by small RNA sequencing (sRNA-Seq) of different poplar tissues during stem development, and its function was verified by the transgene verification. Our findings showed that miR390 has a novel function in poplar stem elongation and plant height growth.

## Results

### Identification and expression profiles of miRNAs during stem development

**Figure 1:**
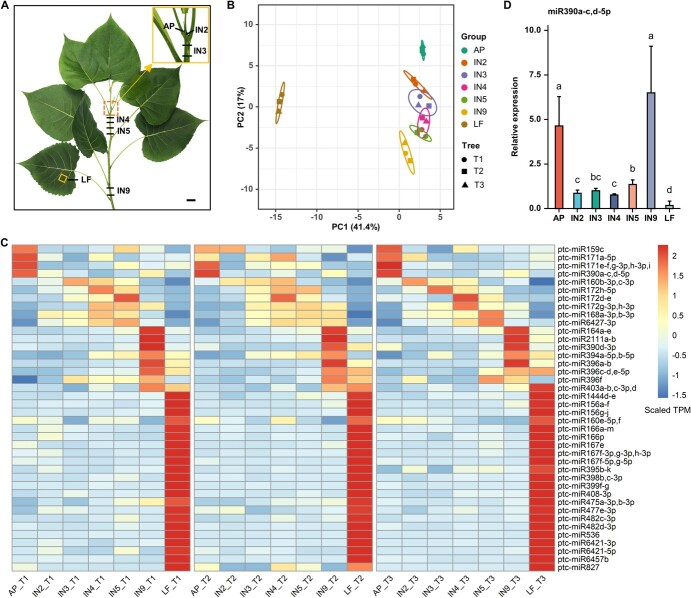
Samples for small RNA sequencing and differentially expressed miRNA during stem development in *P. deltoides* I-69 *×* I-63. **A** Sampling of AP, LF, IN2, IN3, IN4, IN5, and IN9 for sRNA-Seq. The internode samples were collected in length of 2 mm. The scale bar is 2 cm. **B** PCA of miRNAs in AP, IN2-IN5, IN9, and LF samples. Tree (T) 1 to T3 represent the three biological replications. **C** Heat map of 17 differentially expressed miRNA during stem development and 23 highly expressed miRNA in LF. T1 to T3 in the sample names represent the three biological replications of the samples. Expression amount is plotted in scaled TPM using the pheatmap package in R. **D** Accumulation of the mature miR390a-c and miR390d in different samples by RT-qPCR. Data are expressed as mean ± SD (*n* = 3). Statistical significance was determined by one-way ANOVA. Significant differences between means (Duncan, *P* < 0.05) are indicated by lower-case letters above the bar.

To investigate differentially expressed miRNAs during the primary and secondary growth stages of stem development, apex (AP), internode (IN) 2-IN5, and IN9 of 9-year-old *Populus deltoides* I-69 *×* I-63 (hereinafter referred to as *P. deltoides*) were selected as samples for sRNA-Seq (Fig. 1A; Fig. S1, see online supplementary material) according to the transcriptome analysis results in *Populus trichocarpa* [15]. Leaf blades (LF) was used as the control. A total of 9 207 679 to 13 121 893 clean reads were obtained from 21 sRNA libraries (Table S2, see online supplementary material). Principal component analysis (PCA) based on the expression values of miRNAs (Dataset S1, see online supplementary material) indicated clear separations of AP, IN9, and LF from IN2-IN5 samples (Fig. 1B). A total of 75 miRNAs were differentially expressed between sample comparisons (Dataset S2, see online supplementary material). By clustering miRNA expression patterns into seven groups within each biological replicate using k-means clustering, 40 differentially expressed miRNAs with consistent expression patterns among the three biological replicates were obtained (Fig. 1C). Among the differentially expressed miRNAs, four (including miR159c, miR171a-5p, miR171e/f/g-3p/h-3p/I, and miR390a-c/d-5p), six (including miR160b-3p/c-3p, miR172h-5p, miR172d-e, miR172g-3p/h-3p, miR168a-3p/b-3p, and miR6427-3p), and seven miRNAs (including miR164a-e, miR2111a/b, miR390d-3p, miR394a-5p/b-5p, miR396a/b, miR396c/d/e-5p, and miR396f) were highly expressed in AP, the transition from primary growth to secondary growth (IN2-IN5), and IN9, respectively. These miRNAs are potentially important in regulating stem development. miR390a-c/d-5p had high expression levels in AP and were further studied. In *Populus* genome, four *MIR390* genes (*MIR390a-d*) produced five miR390 mature sequences (miR390a-c, miR390d-5p, and miR390d-3p). Among the sequences, the mature sequences of miR390a-c and miR390d-5p were identical. With a focus on highly expressed miRNAs in AP, the expression pattern of mature miR390a-c/d-5p was validated by RT-qPCR, which also exhibited high expression in the AP samples (Fig. 1D).

### miR390b is ubiquitous in poplar

**Figure 2:**
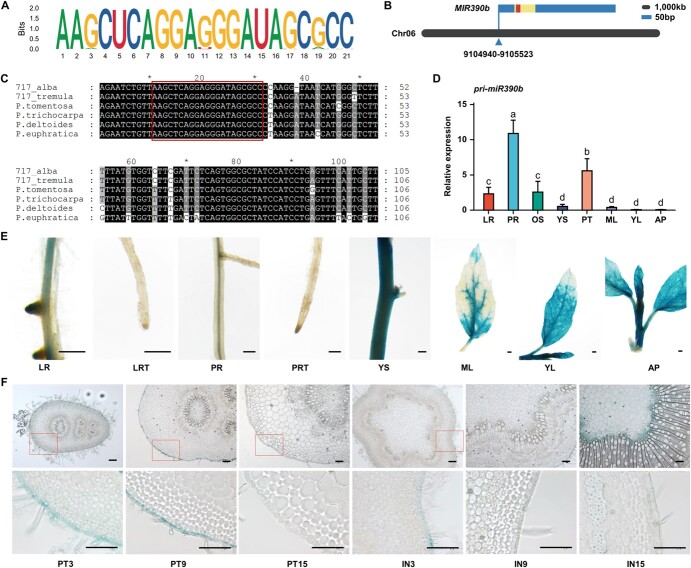
Sequence conservation and expression pattern of miR390b. **A** Conservation of miR390 mature sequences derived from 163 land plant species. The height of the capital letter at each position in the sequence logo represents the degree of conservation. **B** Localization of *MIR390b* gene in the poplar genome. The blue box indicates the full-length of *MIR390b* gene, the orange box indicates miR390b precursor and the red box indicates mature miR390b. **C** Sequence alignment of miR390b precursor in different poplar species. The red box indicates the sequence of mature miR390b. **D** The expression level of miR390b in different organs of WT by RT-qPCR. AP, apex; LR, lateral root; ML, mature leaf; OS, old stem; PR, primary root; PT, petiole; YL, young leaf; YS, young stem. Data are presented as means ± SD (*n* = 3). Statistical significance was determined by one-way ANOVA. Significant differences between means (Duncan, *P* < 0.05) are indicated by lower-case letters above the bar. **E** and **F** Histochemical staining of transgenic poplars harboring the *GUS* reporter gene driven by the promoter of *MIR390b*. The 6-week to 8-week-old tissue-cultured seedlings (**E**) and 6-week to 8-week-old soil-cultured seedlings (**F**) were used for GUS staining. LRT, lateral root tip; PRT, primary root tip. The scale bars are 500 μm in **E** and 100 μm in **F**.

The miR390 family has 374 members from 163 plant species [34], of which 306 members share the same mature sequence (5’-AAGCUCAGGAGGGAUAGCGCC-3′, as the standard mature sequence of miR390), as shown in sequence logo (Fig. 2A). Among the sequences, the mature sequences of miR390a-c/d-5p in poplar were consistent with the standard sequence. RT-qPCR of the primary transcripts revealed four *MIR390* genes with distinct expression patterns in *P. deltoides*. Pri-miR390a/b/d were highly expressed in AP and IN9, while pri-miR390c was specifically expressed in IN9 (Fig. S2, see online supplementary material).

In this study, miR390b was taken as the object. Based on the miR390b precursor sequence, the full length of the miR390b primary transcript was obtained by 5’ RACE and 3’ RACE. Both the full-length transcript and the corresponding DNA sequence of *MIR390b* were 584 bp, indicating that there is no intron in *MIR390b* of *P. deltoides* (Fig. S3, see online supplementary material). The transcription start site was located 89 bp upstream of the mature miR390b, and *MIR390b* was localized on chromosome 06 (Chr06) of the *P. deltoides* genome (Fig. 2B). Although the precursors of miR390b were less conserved among different poplar varieties, all precursors produced identical mature sequences (Fig. 2C). RNA from different tissues of *Populus tremula* × *Populus alba* INRA clone 717 1-B4 (hereinafter referred to as 717 hybrid poplar) was extracted and assayed for quality (Fig. S4, see online supplementary material). The expression level of miR390b was higher in primary root (PR) and petiole (PT) of 717 hybrid poplar, and lower in young stem (YS) and mature leaf (ML), especially in young leaf (YL) and AP (Fig. 2D).

To further understand the expression pattern of *MIR390b*, a β-glucuronidase (GUS) construct driven by the promoter of *MIR390b* was transformed into wild-type (WT) 717 hybrid poplar. GUS staining was detected in AP and YL of 6-week to 8-week-old tissue-cultured seedlings as well as in YS, and also in the main veins of ML, primordia of LR, and vascular tissue of LR and PR (Fig. 2E). Furthermore, weak GUS staining was detected in the primary root tip (PRT) and no staining in the lateral root tip (LRT) (Fig. 2E). In 6-week to 8-week-old soil-cultured plants, GUS staining was detected in epidermal trichome and epidermal cells of the third petiole (PT3) as well as in the primary xylem vessels (Fig. 2F). The expression of miR390b was also observed in the epidermal cells of PT9, but not in PT15 (Fig. 2F). From IN3 to IN9, the expression of miR390b in the epidermal cells of the stem gradually decreased and was eventually undetectable (Fig. 2F). With the transition of stem secondary growth, the expression of miR390b gradually increased in the primary xylem and even in the medullary rays of IN15 (Fig. 2F). The expression of miR390b in tissues continuously changed in a gradual manner with the growth of poplar, reflecting its spatiotemporal specificity.

### Overexpression of miR390b promotes stem elongation in poplar

**Figure 3:**
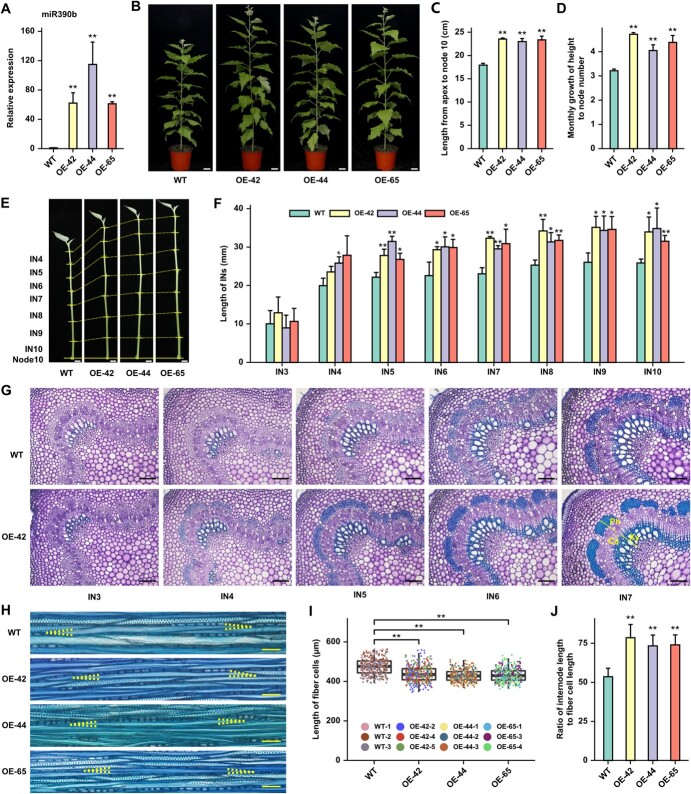
Overexpression of miR390b promotes stem elongation in poplar. **A** The expression levels of mature miR390b in the leaves of 4-week-old tissue-cultured seedlings of WT and lines overexpressing miR390b (OE-miR390b). *PtActin* acted as the endogenous control gene. **B** Morphologic phenotype of WT and OE-miR390b lines. Compared with WT, the OE-miR390b lines exhibited higher plant height in the greenhouse. The scale bars are 5 cm. **C** and **D** Statistical analysis of the length from apex to node 10 and the monthly growth of height to node number of WT and OE-miR390b lines. **E** The morphology observation of WT and three OE-miR390b lines from apex to node 10. The yellow solid lines indicate the positions of the nodes, and the nodes connected by the yellow dotted lines are the corresponding nodes between WT and OE-miR390b lines. The scale bars are 1 cm. **F** Internode length of WT and three OE-miR390b lines from IN3 to IN10 in **E**. **G** Histological analyses of vascular tissue stained with 0.05% toluidine blue. Transverse sections were taken from IN3 to IN7 of WT and OE-42. Ca, cambium; Ph, phloem; Xy, xylem. The scale bars are 100 μm. **H** Histological staining of IN8 longitudinal sections of WT and OE-miR390b lines. The yellow dotted lines indicate the two ends of the xylem fiber cells. Scale bars correspond to 50 μm. **I** and **J** Statistical analysis of the length of IN8 fiber cells in **H**. Data are shown as mean ± SD (*n* = 3). Asterisks indicate statistically significant differences compared with WT by Student’s *t-*test (^*^P < 0.05, ^**^P < 0.01).

To determine whether miR390 participates in the AP development of poplar, WT 717 hybrid poplar was transformed with the vector overexpressing (OE) miR390b for phenotype observation and identification (Fig. S5A**,** see online supplementary material). In the nine independent OE-miR390b lines obtained, the expression levels of miR390b were up-regulated in leaves (Fig. S5B and C, see online supplementary material). Among them, three OE-miR390b lines (i.e. OE-42, OE-44, and OE-65) with the significantly up-regulated expression levels of the precursor and mature miR390b were selected for the subsequent experiments (Fig. 3A, Fig. S5D, see online supplementary material). After WT and OE-miR390b lines were moved into the greenhouse for growth, it was found that the OE-miR390b lines were higher than the WT plants (Fig. 3B). The growth data of plants were measured at a fixed time every week, and the results showed that the lengths from apex to node 10 of OE-miR390b lines were about 30% longer than that of WT (Fig. 3C). Although there was no consistent significant difference in the height growth and node number increment each month for OE-miR390b lines compared to that of WT (Fig. S6A and B, see online supplementary material), the ratio of monthly growth of height to node number was significantly increased (Fig. 3D). In addition, the significant increase in the ratio of plant height to node number in the OE-miR390b lines was caused by a significant increase in plant height rather than an increase in the total node number of the whole plants (Fig. S6C–E, see online supplementary material). To be specific, each IN length of OE-miR390b lines from IN5 to IN10 was significantly longer than that of WT (Fig. 3E and F), without significant differences in base diameter and IN diameter (Fig. S6F and G, see online supplementary material). There were no significant differences in leaf morphology, leaf area, and leaf aspect ratio between the OE-miR390b lines and WT (Fig. S7A–C, see online supplementary material). In addition, no significant differences were observed in root morphology, root dry weight, and root fresh weight (Fig. S7D–F, see online supplementary material).

The IN lengths of poplar trees are determined by two factors: cell number and cell length along the longitudinal axis of the stem. Based on said factors, the development of vascular tissue in soil-cultured plants were observed in transverse sections, and the lengths of xylem fiber cells (which had undergone cell wall thickening) were measured in longitudinal sections (Fig. 3G and H). The vascular development state of IN4 in OE-miR390b lines was essentially similar to that of IN6 in WT, and secondary growth of xylem fiber cells had occurred in IN7 of all OE-miR390b lines and WT (Fig. 3G). The measurement and statistical analysis of the xylem fiber cell lengths of IN8 revealed that the lengths of xylem fiber cells in OE-miR390b lines were 7–10% shorter compared with that of WT (Fig. 3H and I). However, the relative numbers of IN8 xylem fiber cells in the longitudinal axis were 37–47% higher in OE-miR390b lines than that in WT (Fig. 3J). Such results demonstrate that overexpression of miR390 promoted the stem elongation and plant height and increased the number of xylem fiber cells in the longitudinal axis.

### Genome-wide identification of genes in response to miR390 overexpression in apex tissue

Plant SAM determines the number of cells in the longitudinal direction of stem internodes through the regular production of lateral organs, which subsequently affects the length of INs. To explore the downstream pathway of miR390-mediated stem elongation, transcriptome data from the AP tissues of three OE-miR390b lines and WT were compared. On average, over 75% of clean reads of each sample were specifically aligned to the reference genome of *P. trichocarpa* (Table S3, see online supplementary material). The result of PCA showed that three OE-miR390b lines were clearly separated from WT (Fig. S8, see online supplementary material).

**Figure 4:**
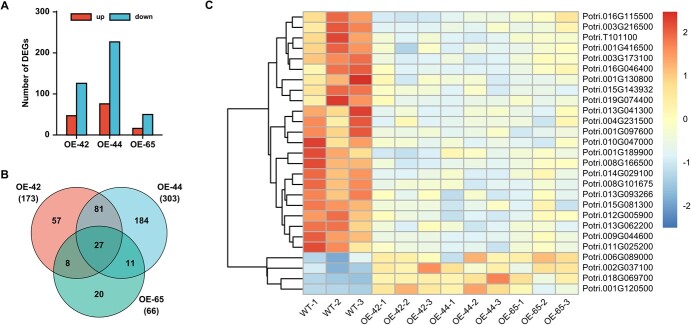
Identification of genes responsive to miR390b overexpression in apex tissue of poplar. RNA-Seq was conducted using the apex tissue of three OE-miR390b lines at 13 weeks of soil culture, the stage of phenotypic stability of stem elongation. The apex tissue of WT was used as the control. **A** The number of up- and down-regulated differentially expressed genes (DEGs) between OE-miR390b lines and WT. **B** The overlapping DEGs among three OE-miR390b lines. The numbers in parentheses indicate the total DEGs at each OE-miR390b line. **C** Clustering of co-expressed DEGs in three OE-miR390b lines. The color code represents the normalized expression level of log10 FPKM. High FPKM values are in red and low FPKM values are in blue. The numbers 1, 2, and 3 represent three biological replicates.

By comparing the gene expression levels between OE-miR390b lines and WT (Dataset S3, see online supplementary material), there were 173, 303, and 66 differentially expressed genes (DEGs) in OE-42, OE-44, and OE-65, respectively, of which 47, 76, and 16 genes were up-regulated and 126, 227, and 50 genes were down-regulated, respectively (Dataset 4, see online supplementary material; Fig. 4A), indicating that over 70% of DEGs in each OE-miR390b line exhibited down-regulated expression in response to overexpression of miR390b. Venn diagram shows that a total of 388 DEGs were identified in three OE-miR390b lines, but they shared only 27 DEGs in common (Fig. 4B; Table S4, see online supplementary material). The heat map shows that four DEGs were up-regulated and 23 DEGs were down-regulated in three OE-miR390b lines (Fig. 4C). Such results indicate that the overexpression of miR390b in poplar affects the expression levels of related genes and mainly plays a negative regulatory role.

### *TAS3.1* and *TAS3.2* are the target genes of miR390 in poplar

**Figure 5:**
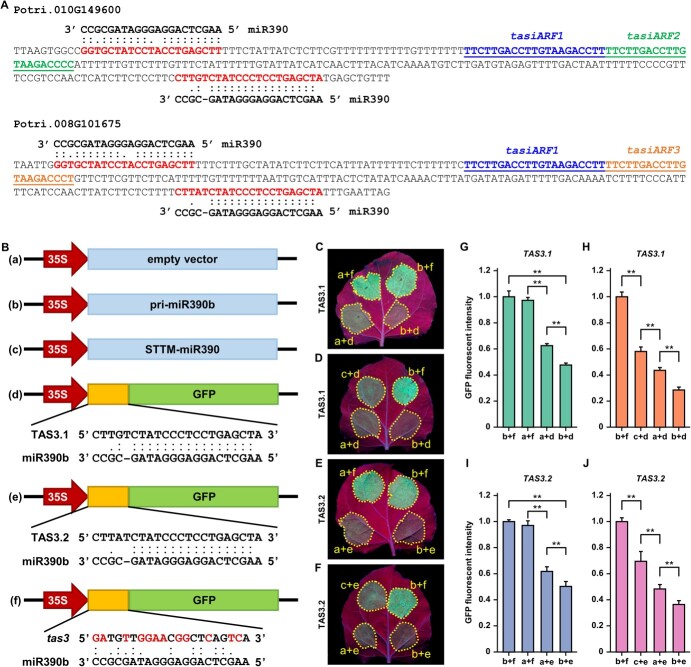
Validation of direct cleavage of *TAS3.1* and *TAS3.2* by miR390 in vivo. **A** Sequence analysis of target genes *TAS3.1* and *TAS3.2.* The red sequences indicate target sites (TSs) complementary to miR390; the blue underlined sequences indicate *tasiARF1*; the green underlined sequence indicates *tasiARF2*; and the orange underlined sequence indicates *tasiARF3*. (**B**) The structure schematic diagram of vectors used in the tobacco transient co-expression. **a**, empty vector; **b**, OE-miR390b vector; **c**, STTM-miR390 vector; **d**, *TAS3.1*–3’TS-GFP vector of overexpression of a GFP fusion protein carrying the target site at the 3′ proximal end of *TAS3.1* gene; **e**, *TAS3.2*–3’TS-GFP vector of overexpression of a GFP fusion protein carrying the TS at the 3′ proximal end of *TAS3.2* gene; **f**, vector of overexpression of a GFP fusion protein carrying the 12 nucleic acid mutation sites of *TAS3* gene; pri-miR390b, primary transcript of miR390b; STTM, short tandem target mimic; *tas3*, mutant of *TAS3* gene. (**C**–**F**) Co-injection of *Agrobacterium tumefaciens* harboring the different vector combinations under the abaxial epidermis of the leaves of *Nicotiana benthamiana*. b + d and b + e are experimental groups, and the other combinations are negative controls. The yellow dotted lines indicate the co-injection areas. **G** and **H** Quantitative analysis of GFP fluorescence intensity in **C** and **D**. **I** and **J** Quantitative analysis of GFP fluorescence intensity in **E** and **F**. Values are expressed as mean ± SD (*n* = 3). Asterisks indicate statistically significant differences using Student’s *t*-test (^**^P < 0.01).

As an endogenous non-coding small RNA, miRNA usually regulates plant growth and development by cleaving mRNA of downstream target genes. Thus, identification of miR390 target genes is of great significance for analysing its function in regulating stem elongation. Four *TAS3* genes, namely *TAS3.1* (*Potri.010G149600*), *TAS3.2* (*Potri.008G101675*), *TAS3.3* (located on Chr18), and *TAS3.4* (*Potri.002G191950*), were identified as candidate targets of miR390 in poplar [34]. Among said genes, *TAS3.2* belonged to one of the DEGs shared by the three OE-miR390b lines (Fig. 4C). The candidate target genes *TAS3.1* and *TAS3.2* were highly homologous and had highly similar sequence structures (Fig. 5A). Consistent with previous reports [34], there was a G:U wobble base pair of the 11th nucleotide and a mismatch of the 12th nucleotide at the 5′ target site (TS), as well as a mismatch of four nucleotides at the 3′ TS in poplar (Fig. 5A). Based on the complementarity of the two TSs with miR390, and combined with previous reports [19], an assumption was made that the TS of miR390 in poplar may be conservatively located at the 3′ proximal end of the candidate targets *TAS3*.

To verify whether miR390 could directly cleave the 3′ proximal end TSs of the two potential target genes, transient co-expression in *Nicotiana benthamiana* leaves was used (Fig. 5B–J). Compared with three negative control groups, the green fluorescent protein (GFP) fluorescence intensity of co-injection with OE-miR390b + *TAS3.1/TAS3.2*–3′ TS-GFP (i.e. b + d/e) was significantly reduced (Fig. 5C, E, G, and I). However, the GFP fluorescence intensity of co-injection with empty vector + *TAS3.1/TAS3.2*–3′ TS-GFP (i.e. a + d/e) was also obviously less than the two other negative controls. In tobacco, the miR390 family has three members (miR390a-c), of which the mature sequences of miR390b and miR390c are identical to that of poplar miR390a-c and miR390d-5p, and the mature miR390a differs from the poplar miR390 by only one base difference at the 3′ proximal end (Fig. S9, see online supplementary material). Therefore, an assumption was made that tobacco miR390 may cleave vectors carrying the 3′ proximal end TS of potential target genes. As such, the GFP fluorescence intensity of tobacco leaves co-injection with STTM-miR390 + *TAS3.1/TAS3.2*–3′ TS-GFP (i.e. c + d/e) was significantly higher than that of OE-miR390b + *TAS3.1/TAS3.2*–3′ TS-GFP and empty vector + *TAS3.1/TAS3.2*–3′ TS-GFP (Fig. 5D, F, H, and J). The results provide evidence that *TAS3.1* and *TAS3.2* are the target genes of miR390 in poplar.

**Figure 6:**
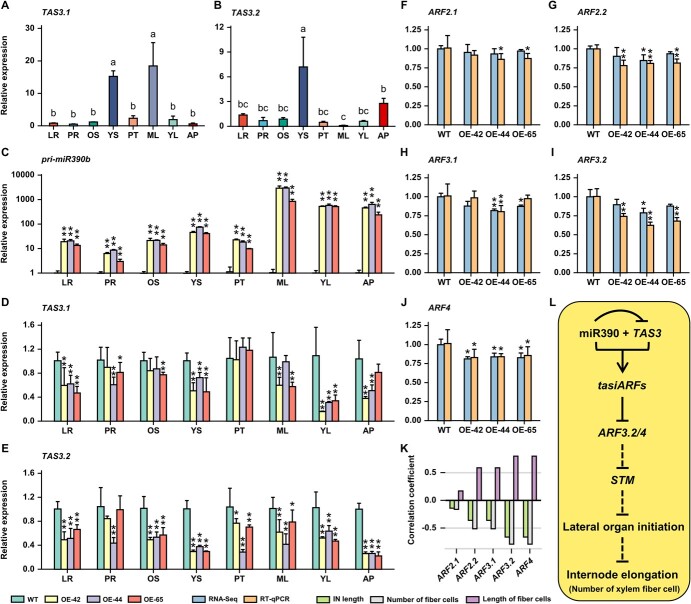
The transcription levels of genes and co-expression analysis between *ARF* expression levels and growth data. **A** and **B** The expression patterns of target genes *TAS3.1* (**A**) and *TAS3.2* (**B**) in different tissues of WT. Statistical significance was determined by one-way ANOVA. Significant differences between means (Duncan, *P* < 0.05) are indicated by lower-case letters above the bar. **C**–**E** The expression levels of miR390b (**C**) and target genes (**D** and **E**) in different tissues of three OE-miR390b lines. The WT acted as the control. LR, lateral root; ML, mature leaf; OS, old stem; PR, primary root; PT, petioles; YL, young leaf; YS, young stem; AP, apex. **F**–**J** The transcription levels of *ARFs* in apex tissue were analysed by RNA-Seq and RT-qPCR. Data are expressed as mean ± SD (*n* = 3). Asterisks indicate statistically significant differences using Student’s *t*-test (^*^P < 0.05, ^**^P < 0.01). **K** Correlation analysis between five *ARF* genes and three phenotypes of OE-miR390b lines. The three phenotypes included IN length, number of fiber cells, and length of fiber cells. **L** The proposed model for miR390/*ARF3.2*/*ARF4* regulating stem elongation in poplar. The *tasiARFs* produced by the degradation of *TAS3* by miR390 negatively regulated the expression of *ARF3.2* and *ARF4*, resulting in the up-regulation of the expression of downstream *STM*. *STM* negatively regulated the initiation of lateral organs, resulting in the increase of the number of xylem fiber cells and then stem elongation.

In WT, the expression level of *TAS3.1* was significantly lower in PR and AP, but higher in YS and ML (Fig. 6A). Except in YS and AP, the expression level of another target gene *TAS3.2* was low in other poplar tissues (Fig. 6B). The expression of miR390b was significantly up-regulated in different tissues of OE-miR390b lines, especially in ML, YL, and AP, which was over 100-fold higher than that of WT (Fig. 6C). Target genes *TAS3.1* and *TAS3.2* exhibited different levels of down-regulated expression in OE-miR390b lines (Fig. 6D and E). *TAS3.1* was significantly down-regulated in LR, YS, and YL of three OE-miR390b lines and down-regulated in PR, old stem (OS), ML, and AP of individual OE-miR390b lines (Fig. 6D). Except for the expression in PR of OE-42 and OE-65, which did not exhibit consistently significant differences, *TAS3.2* was significantly down-regulated in all other tissues of three OE-miR390b lines and was more down-regulated in AP (Fig. 6E). Such results indicate that the overexpression of miR390b effectively suppressed the expression levels of target genes *TAS3.1* and *TAS3.2* in poplar, and the suppression effect on *TAS3.2* was more significant in YS and AP.

### Prediction of *ARFs* regulated by miR390-*TAS3* in *Populus*

To predict which *ARFs* were downstream-regulated by miR390-*TAS3* during stem elongation in *Populus*, phylogenetic analysis based on ARF proteins from *Populus* and *Arabidopsis* were conducted, gene expression of the predicted *ARFs* was analysed by RT-qPCR, and correlation analysis between OE-miR390b phenotypes and whole-genome gene expression levels was performed by weighted correlation network analysis (WGCNA). In *P. trichocarpa* genome, a total of 36 genes are annotated as ARF proteins. The full-length of such protein sequences together with ARF2, ARF3, and ARF4 protein sequences from *Arabidopsis* were used to construct a phylogenetic tree (Fig. S10, see online supplementary material). Proteins of Potri.012G106100 and Potri.015G105300 belonged to the same branch as *Arabidopsis* ARF2, indicating the highest similarity, referred to as ARF2.1 and ARF2.2, respectively. Meanwhile, proteins of Potri.004G050150 and Potri.011G059300 belonged to the same branch as *Arabidopsis* ARF3, and Potri.009G011800 and *Arabidopsis* ARF4 belonged to the same branch, referred to as ARF3.1, ARF3.2, and ARF4, respectively. Although Potri.011G059101 was also predicted to be an ARF3/4 [34], it was omitted in the next study because of the distant evolutionary relationship with *Arabidopsis* ARF3/4. The expression levels of five *ARFs* in poplars were detected by RT-qPCR under the effect of overexpression of miR390b. The results showed that the transcription levels of *ARF2.2*, *ARF3.2*, and *ARF4* were significantly repressed in three OE-miR390b lines (Fig. 6G, I, and J). Although the expression levels of *ARF2.1* and *ARF3.1* were significantly repressed only in individual OE-miR390b lines, the expression levels in other lines also exhibited a down-regulation trend (Fig. 6F and H). The expression trends of *ARF* genes in RNA-Seq data were basically consistent with the results of RT-qPCR (Fig. 6F–J). The co-expression relationship between the gene expression levels from RNA-Seq and multiple phenotypic data of OE-miR390b lines was speculated through the correlation coefficient, and the downstream key *ARFs* of miR390 promoting stem elongation were screened. Given the negative regulatory effect of miR390 on *ARFs*, *ARFs* that were negatively correlated with IN length and the number of fiber cells, and positively correlated with the length of fiber cells were focused on. The five *ARF* gene*s* of poplar were distributed in three modules, *ARF3.2* and *ARF4* in the pink module were most strongly correlated with the above three phenotypes (Fig. 6K; Fig. S11, see online supplementary material). The results reveal that *ARF3.2* and *ARF4* responded to the overexpression of miR390b and cooperated with miR390 to participate in the stem elongation in poplar.

## Discussion

### Identification of microRNAs involved in poplar stem development

sRNA-Seq was performed on APs and INs from 9-year-old *P. deltoides*, with LF samples serving as controls. A total of 17 known miRNAs were differentially expressed during stem development (Fig. 1C), suggesting their potential roles in AP and vascular tissue development. Specifically, miR171 was expressed and enriched in AP, which is consistent with the previous report on *Arabidopsis* [35]. The miR171 function of regulating SAM development by repressing target genes *HAIRY MERISTEMs* (*HAMs*) has been demonstrated in several species [36–38]. In the present study, miR164 was enriched in IN9 of *Populus*. A previous study reported that overexpression of its target gene *NAC* (*NAM*, *ATAF*, and *CUC*) in *Arabidopsis* delayed stem elongation [39]. miR396, also enriched in IN9, has been reported to be involved in regulating internode elongation through the mediation of the target genes *GRF* in several species [40–42]. Furthermore, miR172, with relatively high expression in IN3 and IN4, has been reported to regulate internode elongation during the reproductive stage in rice [43]. Therefore, an assumption was made that differentially expressed miRNAs in AP or stem may be involved in the regulation of growth and stem development in *Populus*. The mature sequence of miR390a-c and miR390d-5p were specifically abundant in AP and IN9. The results of GUS histochemical analysis further reveal the high activity of the miR390b promoter in AP (Fig. 2E). Regulatory pathways consisting of miR390 and its downstream target genes *TAS3* and *ARFs* have been reported in various aspects of plant growth and development, as well as in response to biotic and abiotic stresses [21–32]. Although miR390-*TAS3-ARFs* module has been reported to be involved in the regulation of lateral root growth [33], so far, miR390 function has been less studied in poplar. Poplar is one of the main sources of timber and has an important economic value. However, the functional research of miR390 in poplar stem development remains relatively limited. A series of findings in this study showed that overexpression of miR390b affected the plant height and internode length in poplar. We discovered a novel function of miR390b in poplar involved in regulating stem elongation and affecting woody biomass production, which has important implications for the field of tree biotechnology.

### The regulation of stem length by miR390-*TAS3* in poplar is related to the activity of shoot apical meristem

In the OE-miR390b lines, the trend in the expression level of mature miR390b was consistent with the trend in the number of DEGs (Figs 3A and 4B). It is worth noting that the three OE-miR390b lines showed low overlap of DEGs. There could be several reasons. The three lines are derived from three independent transgenic events. Changing the expression level of mature miR390b in the three lines result in less than 2-fold of change in the expression of the target *ARF*s, which might lead to subtle differences in their downstream network in regulation of organogenesis. In addition, collecting the whole apex as the sample for RNA-Seq might exclude some potential DEGs with high cell type-specific expression. As one of the overlapping 27 DEGs, *TAS3.2* was significantly down-regulated in the majority of tissues of three OE-miR390b lines, suggesting that *TAS3.2* may be a major target gene for stem elongation in *Populus*. Combined with the expression levels of the downstream *ARF3.2* and *ARF4* and correlation analysis with the phenotype of OE-miR390b lines, we come to the hypothesis that the conserved miR390b-*TAS3-ARF*s regulatory pathway may mediate stem elongation in poplar.

It is well known that the increase of bioactive GA level can promote the elongation and growth of plant stems. In *Populus*, several reports have verified that overexpression of GA20 oxidase, a key enzyme for producing bioactive GA, can significantly increase plant height and stem length [4, 44–46]. However, in our study, the increase in stem internode length was not positively correlated to the length of xylem fiber cells (Fig. 3), which is not the same scenario as the previous reports [4, 5, 45]. Furthermore, the pleiotropic effects of elevated GA leading to smaller leaf area and lower root weight in poplar [44–46] was also not observed in the OE-miR390b lines (Fig. S7, see online supplementary material). Therefore, although the OE-miR390b lines resulted in internode elongation, GA signaling pathway may not be directly correlated.

Plant SAM continuously generates new organs in an orderly manner, and the daughter stem cells generated by directed cell division are the source of the vascular cambium. The speed at which SAM produces lateral organs directly determines the number of procambial cells in the stem, which in turn affects the number of vascular cells along the longitudinal axis of the stem internode. It is hypothesized that miR390b-*TAS3*-*ARFs* regulatory pathway affects the stem elongation growth of poplar may be related to SAM development. In a recent study, *ARF3* and *ARF4*, mediated by the histone deacetylase HDA19, repressed *SHOOTMERISTEMLESS* (*STM*) expression and promoted the initiation of reproductive primordia [47]. Down-regulation of *STM* is also an early marker for the formation of leaf primordia in SAM [48]. Moreover, *STM* is specifically expressed in SAM and is a significant regulatory factor in the maintenance of meristem stem cells [49]. In the present study, the down-regulated expression of *ARF3.2* and *ARF4* in AP may affect the expression of *STM* and thereby disturb the homeostasis of cell division and cell differentiation in AP. The disruption may also have led to an increase in the cell number between leaf primordia in the longitudinal direction, or an increase in the cell number of the procambium and/or the precursor cells thereof (Fig. 6L). *STM* was not identified as a differentially expressed gene in the present RNA-Seq analysis, which could be due to its fine-tune regulation or cell-type specificity. The development of specific cellular markers for poplar AP will facilitate precise identification of the cell types involved in miR390b promoting cell division in AP. Additionally, the rapid development of single-cell RNA-Seq technology will also provide new solutions for cell type-specific gene expression analysis.

### Functional diversification of miR390-*TAS3* in *Populus*

Multiple studies have shown that miR390-*TAS3* is involved in regulating LR growth in a variety of plants [22, 23, 29], including *Populus* [33]. However, in the present study, no significant difference was observed in LR development in OE-miR390b plants compared with WT. Similarly, overexpression of *Os*ta-siR2141 and *OsTAS3a* both resulted in the down-regulation of *OsARF3* in rice. Compared with WT, root morphology was not significantly different in plants overexpressing *Os*ta-siR2411, whereas overexpression of *OsTAS3a* significantly increased LR formation [30, 50]. Therefore, an assumption was made that miR390-*TAS3* may be involved in regulating different aspects of plant growth and development in different species and even different varieties.

*ARF3* and *ARF4* were the positive regulators that maintain cambial activity [51]. In the present study, the expression levels of *TAS3.1* and *TAS3.2* were higher in YS of 717 hybrid poplar, and both of them were significantly inhibited by miR390b in the OE-miR390b lines. However, no significant changes in the cell morphology of the vascular cambium were observed in said lines. The inhibitory effect of miR390b-*TAS3* on *ARF3.2*/*4* in AP was significant but relatively mild. The cell-type specificity of *TAS3.1* and *TAS3.2* expression in the stems and the intercellular transport of miR390b and *tasiARF*s remain unclear. Even though miR390-*TAS3* can inhibit the expression of *ARF3* and *ARF4* in the cambium, whether its activity is sufficiently suppressed in the cambium needs to be further investigated.

In summary, overexpression of miR390b can promote stem elongation and height growth in 717 hybrid poplar. Our results suggest that the miR390b-*TAS3.1*/*TAS3.2*-*ARF3.2*/*ARF4* pathway is involved in regulating these biological processes. The involvement of miR390b-*TAS3* in regulating tree height growth adds a new function to the miR390 family in regulating plant growth and development, which is also of great significance for tree biotechnology research.

## Materials and methods

### Plant material and growth conditions

For sRNA-Seq, 9-year-old *P. deltoides* clones were grown at a field site in Huazhong Agricultural University (Wuhan, China).

The tissue-cultured seedlings of 717 hybrid poplar and transgenic lines were grown on 1/2MS medium, subcultured by micro-cutting regularly every two months in a tissue culture room with a photoperiod of 16 h light and 8 h dark at 25°C. Two-month-old, robust tissue-cultured seedlings of WT and transgenic plants were transferred to the poplar growth room for 8 weeks with soil culture, then transplanted to 16 × 18 cm large pots and grown in a greenhouse under natural light conditions from 10°C to 35°C. *N. benthamiana* plants were grown in the plant growth room with long-day conditions (16 h light and 8 h dark) at 23°C.

### sRNA-Seq analysis

For sRNA-Seq, samples of the AP (contains SAM), stem segments of IN2-IN5 (no obvious secondary growth occurred in the vascular tissues), IN9 (contains mature vascular tissue), and LF without midrib were collected from one branch (Fig. 1A; Fig. S1, see online supplementary material). Three branches from three independent 9-year-old *P. deltoides* trees (T1, T2, and T3) were selected as three biological replicates, respectively. IN samples were collected in lengths of 2 mm and sample AP was collected from apical buds (Fig. 1A). Total RNA was separately isolated using Trizol reagent (Invitrogen, China). Libraries for sRNA-Seq were generated as described in the NEBNext Multiplex Small RNA Library Prep Set for Illumina after total RNA quality control. The libraries were sequenced on the Illumina HiSeq 2500/2000 platform, and single-end reads with 50 bp were generated.

Clean reads were obtained from raw data after removing low-quality reads, reads with 5′ adapter contaminants, and reads without 3′ adapter or the insert tag. The certain range (18–30 nt) of lengths from the clean reads were mapped to the reference *P. trichocarpa* v3.1 genome by Bowtie without mismatch. Known miRNAs were identified by aligning clean reads with the miRBase22.1 database (https://mirbase.org/). Unknown sRNA were obtained by excluding (i) non-coding RNA sequences (rRNAs, tRNAs, snRNAs, and snoRNAs) using NCBI (http://www.ncbi.nlm.nih.gov/) and Rfam (http://www.sanger.ac.uk/resources/databases/rfam.html); (ii) repeat sequences using a repeat sequence database (http://www.repeatmasker.org/cgi-bin/WEBRepeatMasker/webcite); and (iii) tags originating from protein-coding genes by mapping to the exon and intron of mRNAs of *Populus*. The potentially novel miRNAs were predicted with miRDeep-P from the remaining unknown sRNA. The abundance of miRNA was generated based on transcripts per million (TPM) values. Differential expression analysis of miRNAs among the samples was performed using R-package DEseq2, with the threshold for significant differential expression of absolute value log2 (fold change) ≥1.0 and adjusted *P* value <0.05. Expression amount is plotted in scaled TPM using the pheatmap package in R. Scaled TPM values were obtained by scaling TPM of each miRNA among samples within each biological replicate, and 0.01 was added to the TPM values for LF samples before scaled.

### Gene cloning and sequence analysis

Total RNA was extracted from the IN9 of 9-year-old *P. deltoides* using the improved 2× CTAB method and reverse transcribed into cDNA in accordance with the manufacturer’s instructions. According to the precursor sequence of miR390b, the full-length transcript of *MIR390b* was obtained from IN9 cDNA of *P. deltoides* by SMARTer® RACE 5′/3′ Kit Components (TaKaRa, China) using the primers listed in Table S1 (see online supplementary material). Subsequently, the full-length sequence of miR390b was amplified from IN9 gDNA of *P. deltoides* using primers miR390b-Full-F/R (Table S1, see online supplementary material).

The sequence conservation of all the members of the miR390 family in land plants was analysed by the online software WEBLOG. Software ClustalX and GeneDoc were used for sequence alignment and editing alignment results, respectively. The precursor sequences of miR390b in *Populus euphratica*, *Populus tomentosa*, and *P. trichocarpa* were downloaded from the online database NCBI (https://www.ncbi.nlm.nih.gov/) and in 717 hybrid poplar from the online database AspenDB (http://aspendb.uga.edu/index.php/databases/spta-717-genome). The full-length sequences of *TAS3* genes and ARF proteins were obtained from the *P. trichocarpa* v3.1 genome (https://phytozome-next.jgi.doe.gov/). The phylogenetic tree for the full-length sequence of ARF proteins was constructed using MEGA5.

### Construction of expression vector and generation of transgenic lines

For the construction of the GUS fusion vector, a 2587 bp promoter fragment upstream of the miR390 coding gene start site was amplified from *P. trichocarpa* gDNA, cloned into the entry vector pDONR201 (Invitrogen, China), and then recombined into the upstream of GUS protein of the expression vector pKGWFS7. For the construction of the overexpression vector, the full length of *MIR390b* was ligated to the entry vector pGWC and then recombined into the overexpression vector pH2GW7, a plasmid carrying the 35S promoter and a hygromycin-resistance gene, using the Gateway LR reaction (Invitrogen, China).

The expression vectors were transformed into *Agrobacterium tumefaciens* and then stably transformed into 717 hybrid poplar by leaf disc transformation [52]. The sequence fragments of reporter genes or/and target genes in the expression vector were amplified by PCR to screen and identify the transgenic positive lines (Table S1, see online supplementary material).

### Gene expression assays

For the quantification of the miR390b precursor, total RNA was extracted from the fourth leaf at the top of the 4-week-old tissue-cultured seedlings of transgenic plants using the Ultrapure RNA Kit (CWBIO, China). For tissue-specific expression quantification of related genes, LR, PR, OS, YS, PT, ML, YL, and AP of 13-week-old soil-cultured plants of transgenic plants were collected. Based on the characteristics of tissue samples, total RNA was extracted from OS and PT using TransZol (TRANS, China), from YS, YL, ML, and AP using the Ultrapure RNA Kit, from PR and LR using the HiPure Plant RNA Mini Kit (Magen, China) according to the manufacturer’s protocols. RNA quality was evaluated using the NanoDrop 2000 spectrophotometer (Thermo Scientific, Wilmington, Delaware, USA) and semi-quantitative RT-PCR.

The transcription levels of genes were detected using RT-qPCR. RNA samples were treated with the gDNA eraser and then reverse-transcribed into cDNA using the primeScript RT reagent Kit (TaKaRa, China) according to the manufacturer’s instructions, with *PtActin* as the reference gene. The RT-qPCR was performed using 2× ChamQ Universal SYBR qPCR Master Mix (Vazyme, China) and gene-specific primer pairs (Table S1, see online supplementary material) on a Roche LightCycler 480 II, according to the manufacturer’s instructions. The assays were performed using three independent biological replicates, each comprising three technical replicates.

### GUS staining

Different tissues of transgenic plants were added with pre-cooled 90% acetone (v/v) and fixed at 4°C for 20 min. The samples were washed twice with potassium phosphate buffer for removing acetone, immersed in X-Gluc solution for treatment at 37°C in the dark for 5–24 hours, and washed with 70% alcohol every 3–4 hours until the WT tissues were completely decolorized. The tissue samples of the cultured seedlings were observed under a stereoscopic microscope (Leica, Germany) and photographed for preservation.

### Length measurement and number estimation of fiber cell

The stem internodes were transversely and longitudinally sectioned using an automatic vibration slicer (Leica, Germany). The sections were stained with 0.05% (w/v) aqueous toluidine blue reagent for lignin characterization, then observed and photographed under a fluorescence microscope (Olympus, Japan).

The length of xylem fiber cells was measured by ImageJ software. A total of 120 fiber cells of each IN8 were selected for measurement, and each line had three independent plants acting as three biological replicates. The number of fiber cells in each IN8 was estimated by the ratio of IN8 length to the average length of fiber cells [53].

### RNA-Seq analysis

To compare gene expression profiles, AP tissue samples (about 5 mm long) of 13-week-old soil-cultured plants of WT and three OE-miR390b lines were collected. Removal of gDNA from total RNA using Recombinant DNase I (RNase-free) (TaKaRa, Japan). The RNA-seq libraries were constructed using NEBNext® UltraTM RNA Library Prep Kit for Illumina® (NEB, Santiago, CA, USA). The Agilent 2100 bioanalyser was used to detect the size of the library insert and the effective concentration of the library was accurately quantified by RT-qPCR The prepared DNA Nano Ball was loaded onto the sequencing chip and the transcriptome was sequenced in Illumina Hiseq PE150 (Frasergen, China). Raw reads were filtered to obtain high-quality clean reads. The quality of clean reads was detected using FastQC software and high-quality reads were mapped to the reference *P. trichocarpa* v3.1 genome using HISAT2 software. The raw read counts of each gene were calculated by HTSeq, followed by identification of DEGs using R-package DEseq2, with the criteria of the absolute value log2 (fold change) ≥1.0 and adjusted *P*-value <0.05.

### Transient co-expression assay

The transient co-expression assay was performed as described previously [54]. The short tandem target mimic sequence of miR390 (STTM-miR390) was synthesized [55]. The TSs of miR390 candidate target genes were cloned into the pMS4v2 vector (between *Xho*I and *Xba*I sites) carrying the GFP coding gene and driving expression using the cauliflower mosaic virus 35S promoter. A mutant TS (*tas3*) that could not be cleaved by miR390 was also cloned into the pMS4v2 for negative control.

The equivalent of the two *A. tumefaciens* (GV3101) suspensions were mixed and co-injected into fully expanded tender leaves of 5-week-old *N. benthamiana*. The GFP signals were observed using a handheld UV analyser 48-72 h after infiltration. The sequence-specific primers for constructing said plasmids in the present experiment are shown in Table S1 (see online supplementary material).

### Statistical analysis

In all experiments of the present study, at least three independent plants with the same age and cultured conditions were used. Excel software (Microsoft) was used for the statistical analysis of the data with at least three biological replicates and three technical replicates. Statistically significant differences were determined by two-tailed and two-sample Student’s *t-*test (*P < 0.05, **P < 0.01), or assessed by analysis of ANOVA followed by Duncan’s multiple comparisons (*P* < 0.05).

## Acknowledgments

This study was supported by the National Natural Science Foundation of China (32171821 and 31770639), Science and Technology Projects of Shennongjia Academy of Forestry, Hubei, China (Grant number: SAF202107), and the Project 2662022YLYJ009 supported by the Fundamental Research Funds for the Central Universities. We are grateful to Professor Feng Li (Huazhong Agricultural University) for providing the vector pMS4v2, and Dr Kebing Du (Huazhong Agricultural University) for supplying the *P. deltoides* clones.

## Author contributions

X.S. and B.Z. designed the project. Q.S., D.T., A.C., Y.M., J.W., Y.C., and J.L. performed the experiments. Q.S. and X.S. analysed the data and drafted the manuscript. Q.S., W.G., X.W., B.Z., and X.S. edited the manuscript.

## Data availability

The sRNA-Seq data and RNA-Seq data have been deposited in the NCBI Sequence Read Archive under BioProject accession numbers PRJNA833047 and PRJNA833269, respectively.

## Conflict of interests

The authors declare that they have no conflict of interest.

## Supplementary data

Supplementary data is available at *Horticulture Research* online.

## Supplementary Material

Web_Material_uhac258
