# Supplementary material for: Overexpression of miR390b promotes stem elongation and height growth in Populus
Source: Hortic Res. 2022 Nov 21;10(2):uhac258. doi: 10.1093/hr/uhac258 (PMC9907050; doi:10.1093/hr/uhac258)
Supplement: Web_Material_uhac258 [file web_material_uhac258.zip › Supplementary Figures S1-S11+Tables S1-S4-2022.10.29-submitted.pdf]

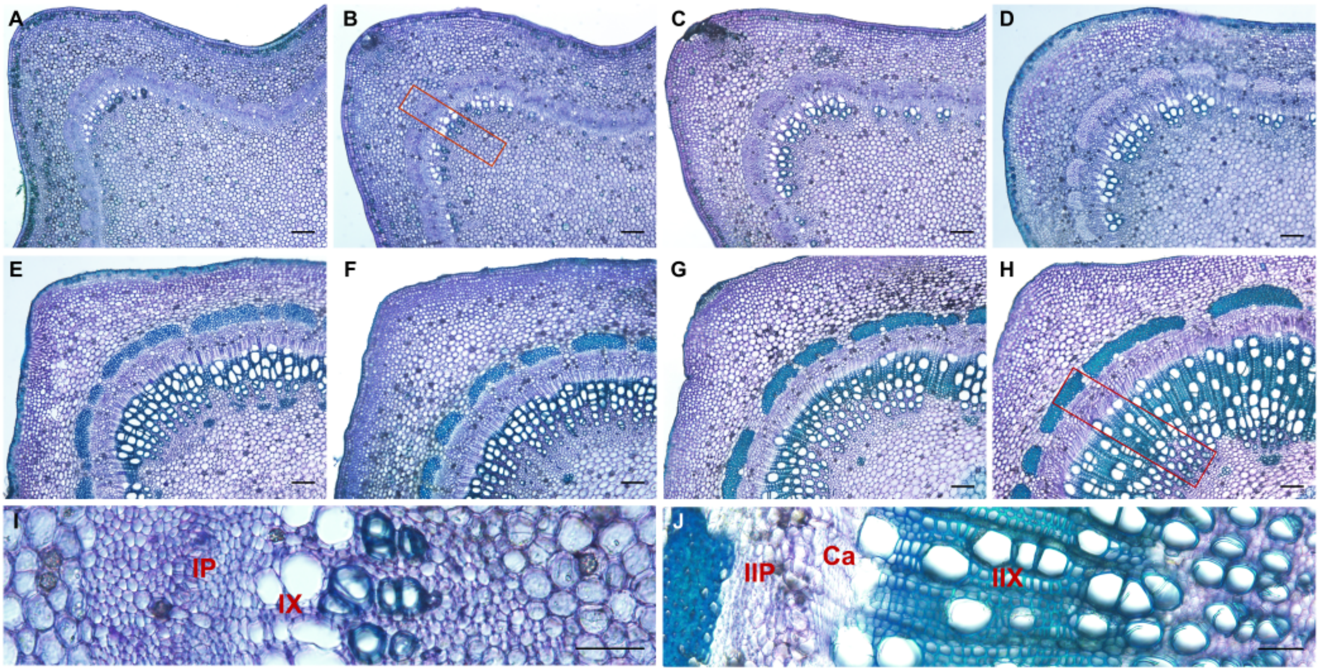

Supplementary Fig. 1 Histological analyses of stem development during primary and secondary growth phases in *P. deltoides*.

Primary growth of stem was observed in internode (IN) 2 (A) and IN3 (B), in which primary xylem and primary phloem were the dominant vasculature. Primary to secondary growth transition occurred in IN4 (C). Secondary growth was observed in IN5 to IN9 (D-H). A mature vascular tissue was formed in IN9 (H), containing cambium, primary and secondary xylem, primary and secondary phloem. Primary xylem (IX) and primary phloem (IP) were shown in magnified view of red box in B (I). Secondary xylem (IIX), secondary phloem (IIP), and vascular cambium (Ca) were shown in magnified view of the red box in H (J). The transverse sections were stained with 0.05% toluidine blue. Scale bars for (A-H) is 100 µm, and that for (I and J) is 50 µm.

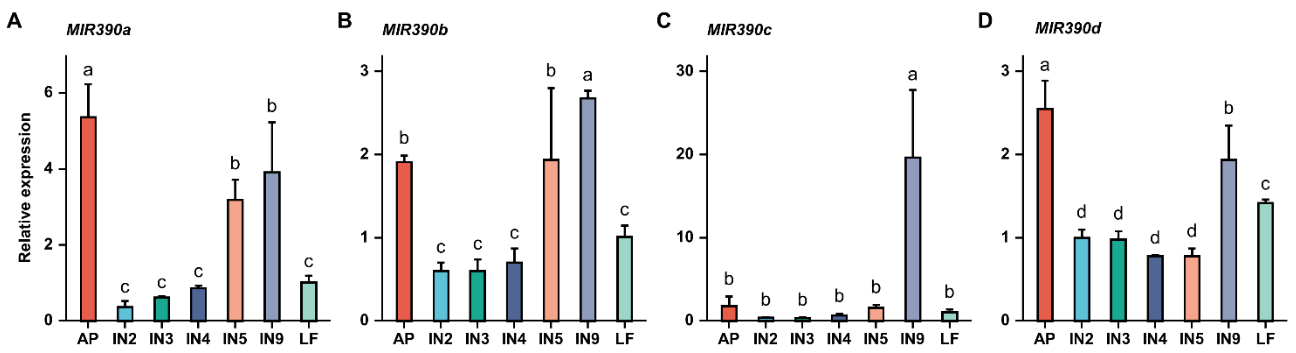

Supplementary Fig. 2 RT-qPCR analysis of the expression of *MIR390s* in *P. deltoides* stem during primary and secondary growth phases.

Expression patterns of *MIR390a* (A), *MIR390b* (B), *MIR390c* (C) and *MIR390d* (D) in apex (AP), internode (IN) 2, IN3, IN4, IN5, IN9 and leaf (LF) samples. The relative expression level was calculated according to the $2^{-\Delta\Delta CT}$ method with *PtActin* reference gene as control. Data are expressed as mean ± SD (n = 3). Experiments were performed with three biological replicates and three technical replicates. Different lower letters replicate significant differences ($P < 0.05$).

A

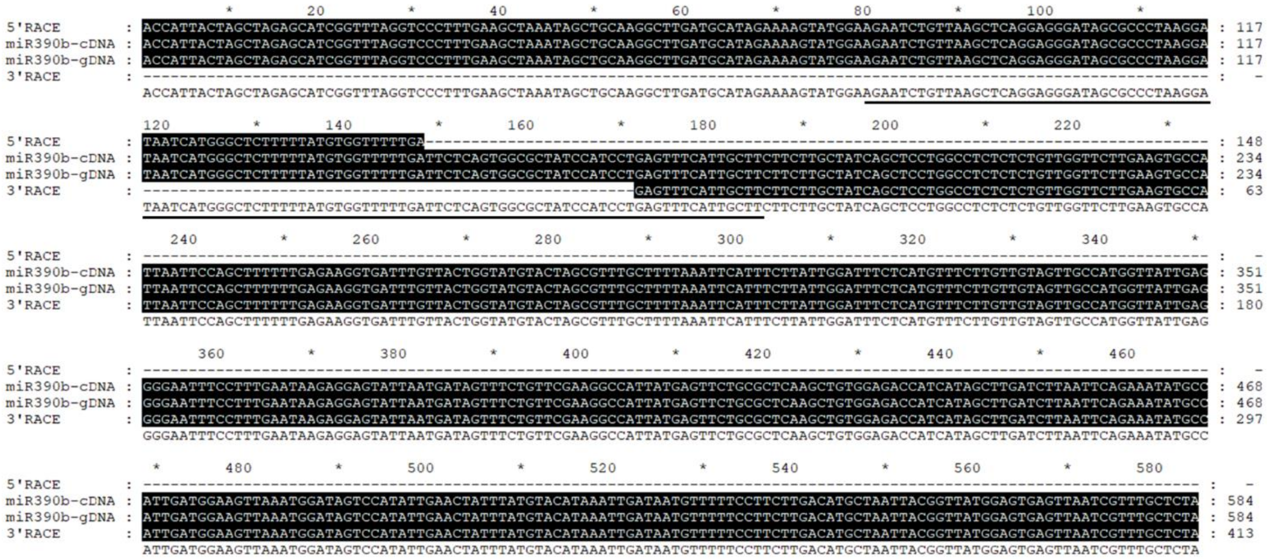

B

>*MIR390b*（584bp）

ACCATTACTAGCTAGAGCATCGGTTTAGGTCCCTTTGAAGCTAAATAGCTGCAAGGCTTGATGCATAGAAAAGTATGGAAGAATCTGTT**AAGCTCAG**
GAGGGATAGCGCCCTAAGGATAATCATGGGCTCTTTTTATGTGGTTTTTGATTCTCAGTGGCGCTATCCATCCTGAGTTTCATTGCTTCTTCTTGCT
ACCAGCTCCTGGCCTCTCTCTGTTGGGTCTTGAAGTGCCATTAATTCCAGCTTTTTTGAGAAGGTGATTTGTTACTGGTATGTACTAGCGTTTGCTT
TTAAATTCATTTCTTATCGGGTTTCTCATGTTTCTTGTTGTAGTTGCCATGGTTATTGAGGGGAATTTCCTTTGAATAAGTGGAGTATTAATGATAG
TTTCTGTTCGAAGGCCATTATGAGTTCTGCGCTCATGCTGTGGAAACCATCATAGATTGATCTTAATTCAGAAATATGCCATTGATGGAAGTTAAAT
GGATAGTCCATATTGAACTATTTATGTACATAAATTGATAATGTTTTTCCTTCTTGACATGCTAATTACGGTTATGGAGGGAGTTAATCGTTTGCTC
TA

Supplementary Fig. 3 Sequence of *MIR390b* gene in *P. deltoides*.

(A) Sequence alignment analysis of miR390b by PCR amplification. 5’/3’RACE, 5’/3’ proximal sequence of miR390b transcript; miR390b-cDNA/gDNA, cDNA/gDNA sequence of *MIR390b* gene. (B) Full-length sequence of *MIR390b* gene (584bp). Green represents the 5’ proximal sequence of miR390b transcript obtained by 5’ RACE; and bule represents the 3’ proximal sequence of miR390b transcript obtained by 3’ RACE. Black underline indicates miR390b precursor; and mature miR390b is in bold.

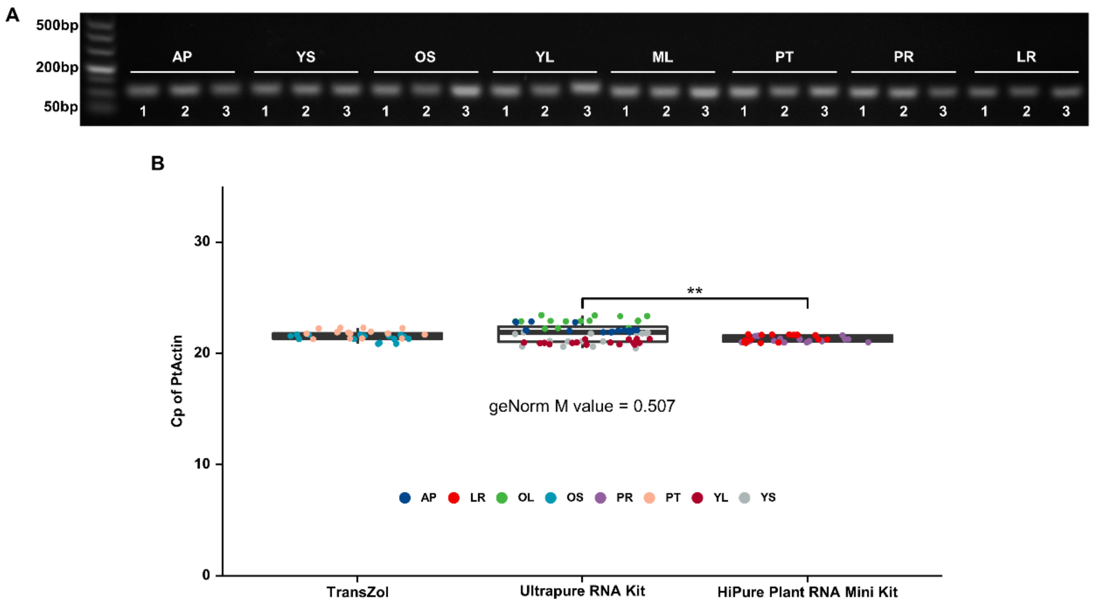

Supplementary Fig. 4 Quality detection of RNA extracted by different RNA extraction kits. Gel electrophoresis image of semi-quantitative RT-PCR analysis of the reference gene *PtActin* (A) and Cp value distribution of the reference gene *PtActin* by qRT-PCR (B). AP, apex; YS, young stem; OS, old stem; YL, young leaf; ML, mature leaf; PT, petiole; PR, primary root; LR, lateral root. All the RNA samples above were from different tissues of 717 hybrid poplar. The numbers 1, 2 and 3 represent three biological replicates. The geNorm M value (cutoff = 1.5) was used to indicate *PtActin* as a stable gene for RT-qPCR analysis. Asterisks indicate statistically significant differences by Student's *t*-test (** $P < 0.01$).

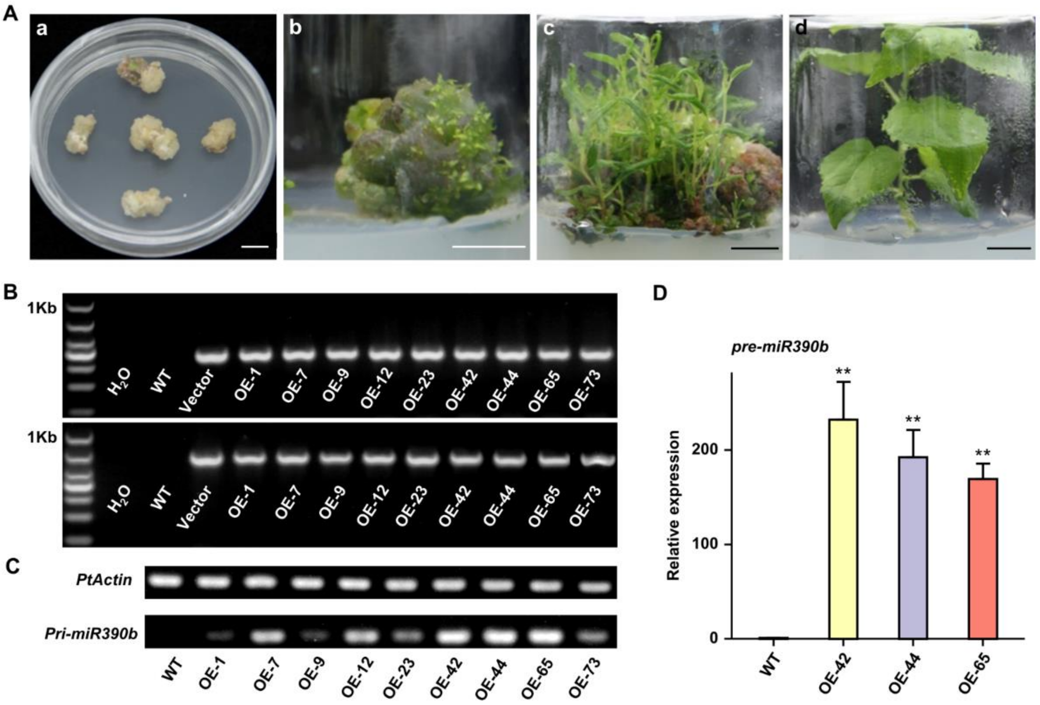

Supplementary Fig. 5 Positive identification of OE-miR390b lines and expression analysis of miR390b precursor.

(A) Generation of overexpressed miR390b (OE-miR390b) lines. a, callus induction; b and c, induction of adventitious buds from callus (b) and shoot elongation (c) in transgenic resistant callus; d, antibiotic-resistant transgenic plant. The scale bars are 1 cm. **(B)** Identification of transgene-positive lines by amplifying the *Hpt II* gene (upper) and the full-length transcript of miR390b coding gene (lower). H_2O and WT were negative controls, while OE-miR390b vector (Vector) was positive control. OE-1 to 73 were positive OE lines. **(C** and **D)** The expression levels of miR390b precursor in leaves were detected by semi-quantitative RT-PCR and RT-qPCR. *PtActin* acted as the internal reference gene. Data are shown as mean ± SD (n =3). Asterisks indicate statistically significant differences compared with WT by Student's t-test (** $P < 0.01$).

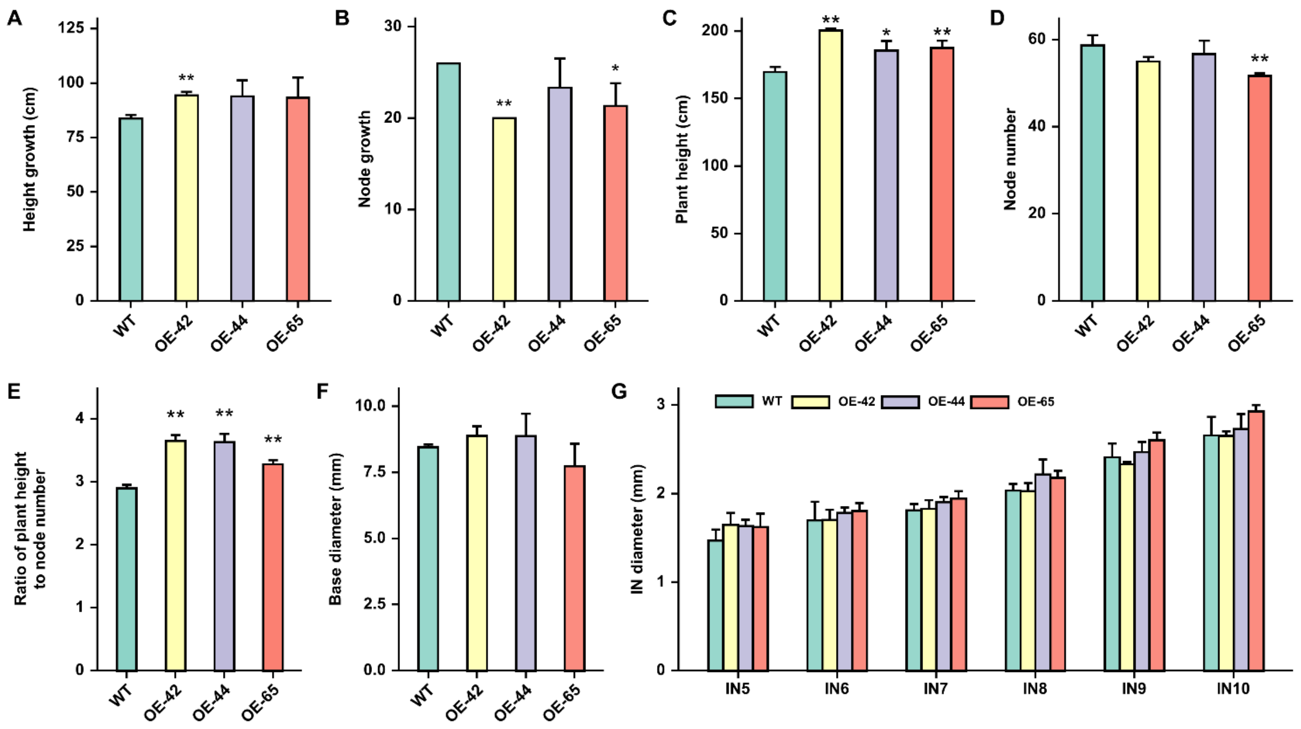

Supplementary Fig. 6 Phenotype observation of stem of OE-miR390b lines.

(A and B) Statistical analysis of monthly height growth (A) and monthly node growth (B) of WT and three OE-miR390b lines. (C and D) Statistical analysis of plant height (C) and total node number of the whole plants. (E) Ratio of plant height to node number of the whole plant. (F and G) Statistical analysis of base diameter (F) and internode (IN) diameter from IN5 to IN10 (G). Above growth data were collected from 13-week-old plants. Data are expressed as means ± SD (n = 3). Asterisks indicate statistically significant differences using Student's t-test (* $P < 0.05$, ** $P < 0.01$).

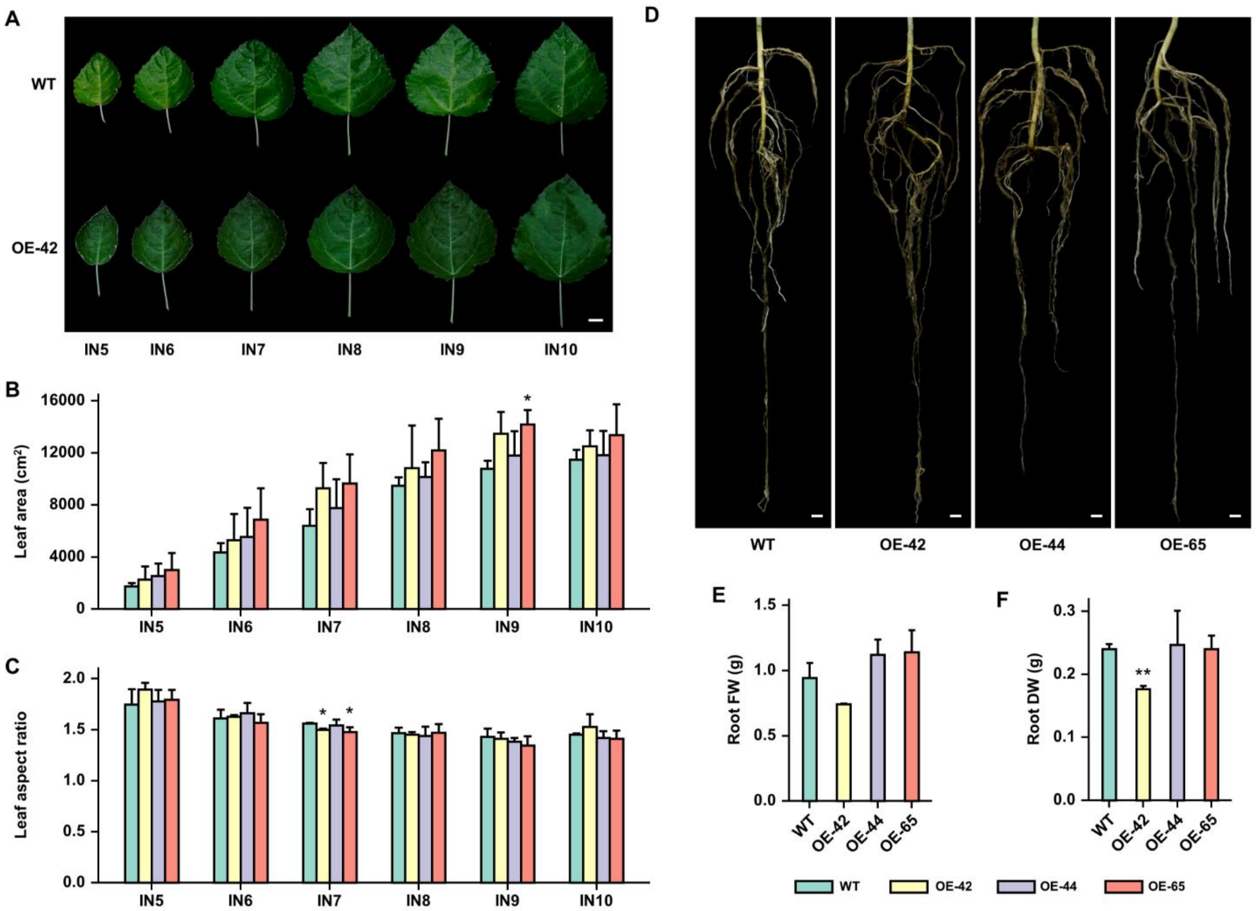

Supplementary Fig. 7 Phenotypic observation of leaves and roots of OE-miR390b lines.
(A) The leaf morphology observation from IN5 to IN10 of WT and OE-42 cultivated in soil. The scale bar is 1 cm. (B and C) Statistical analysis of leaf area (B) and leaf aspect ratio (C) from IN5 to IN10. (D) The root morphology observation. The scale bars are 1 cm. (E and F) Quantification analysis of average fresh weight (FW) (E) and dry weight (DW) (F) of root biomass from three OE-miR390b lines cultivated in soil. Data are expressed as means ± SD (n = 3). Asterisks indicate statistically significant differences using Student's t-test (* $P < 0.05$, ** $P < 0.01$).

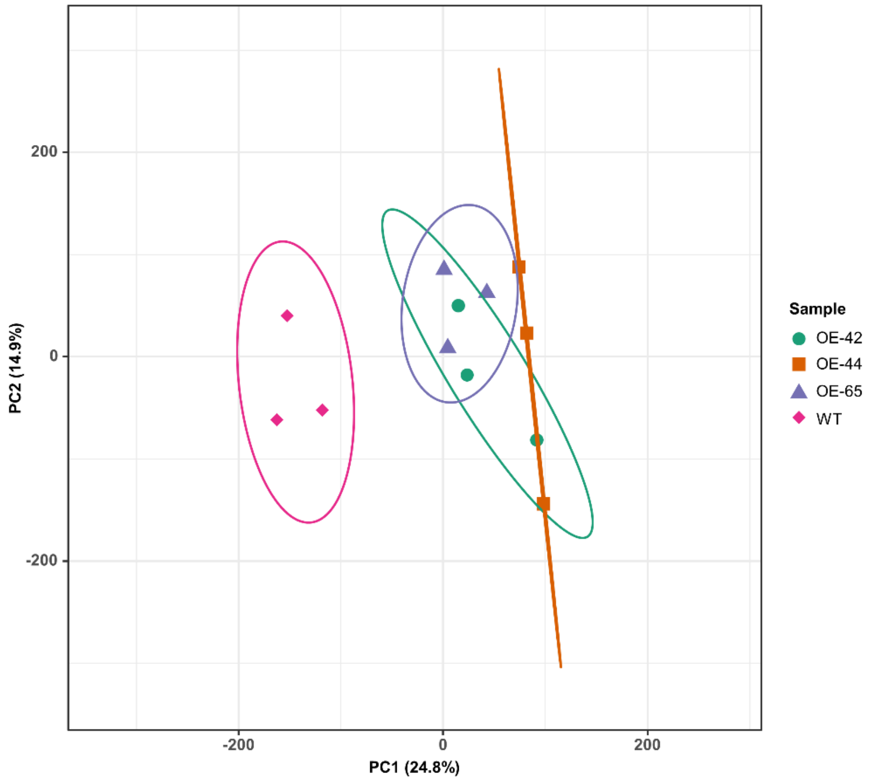

Supplementary Fig. 8 Principal component analysis of global gene expression profiles in three OE-miR390b lines and WT demonstrated a distinct clustering.

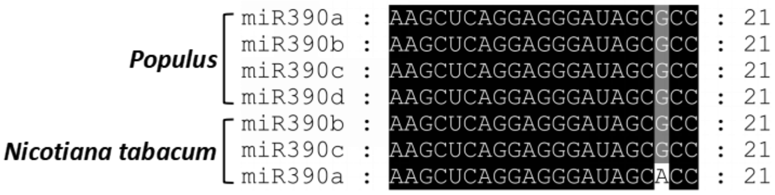

Supplementary Fig. 9 Sequence analysis of mature miR390 in *Populus* and *Nicotiana benthamiana*.

The numbers in the right column indicate length of mature miR390.

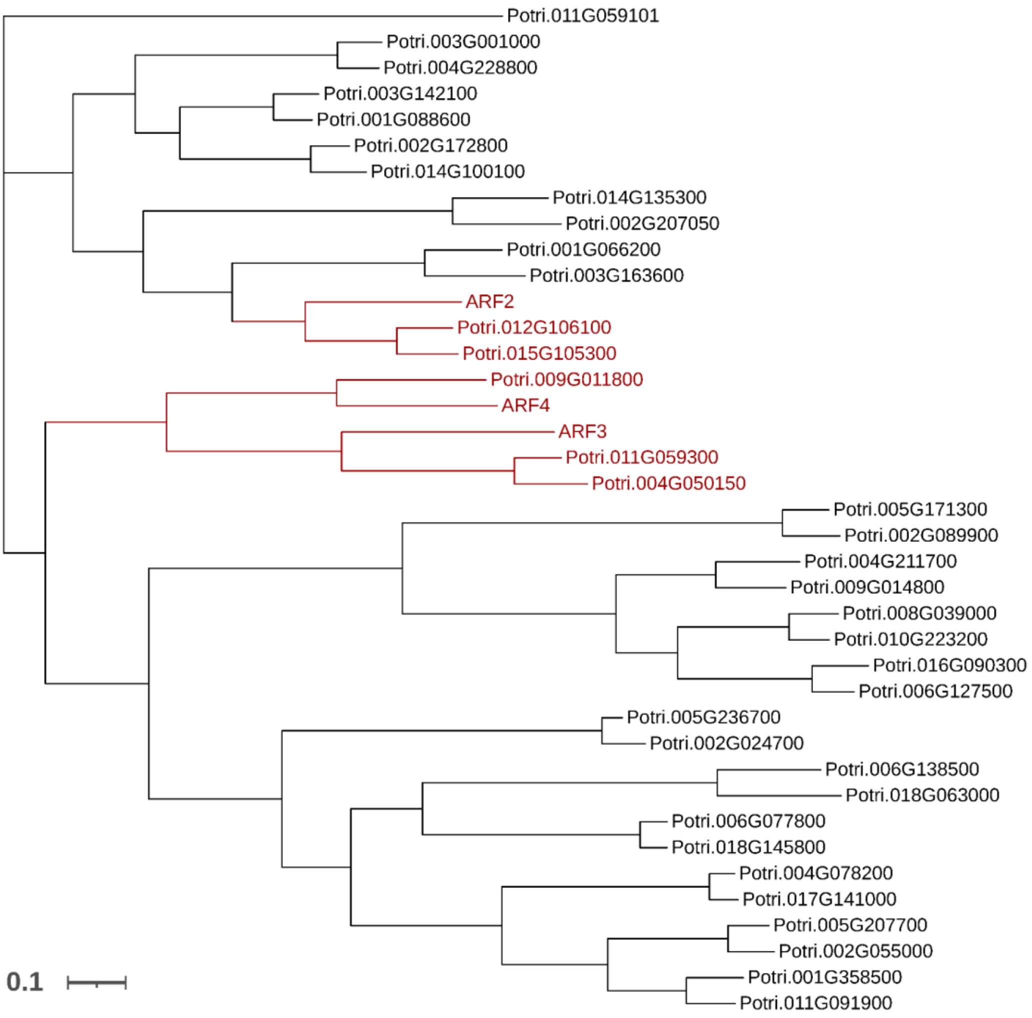

Supplementary Fig. 10 The phylogenetic relationships of all ARF proteins in *Populus* and ARF2/3/4 in *Arabidopsis*.

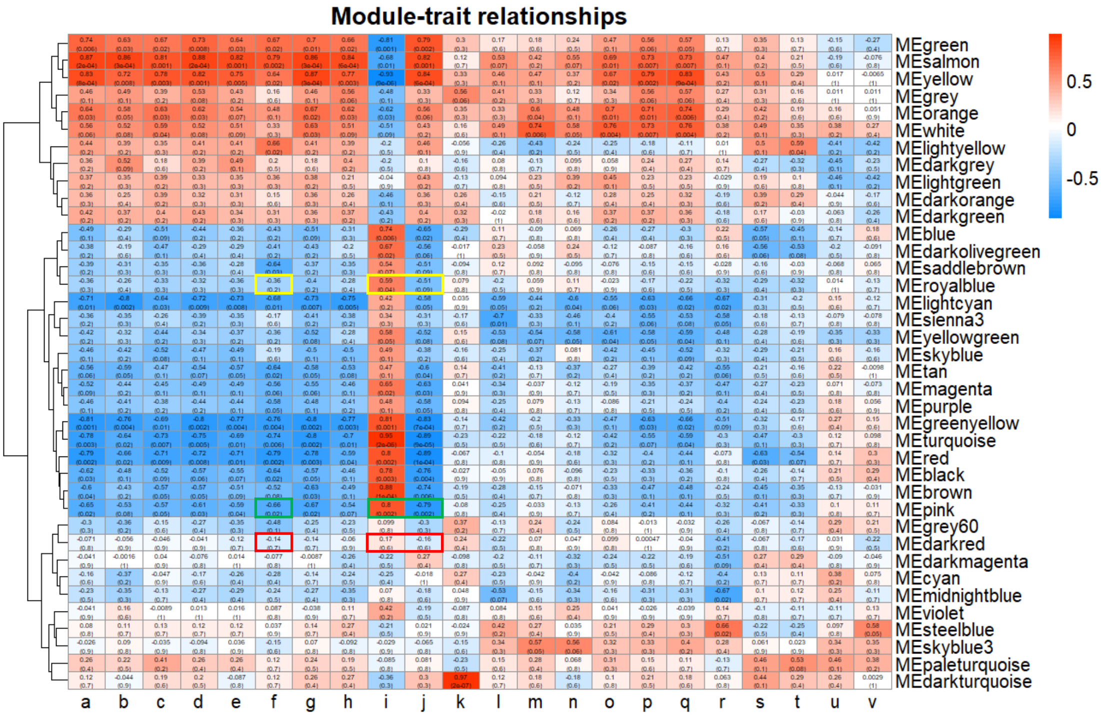

Supplementary Fig. 11 Correlation analysis between whole-genome gene expression values and multiple phenotypes of OE-miR390b lines.

The X-axis indicates the phenotypic data (a-v) of OE-miR390b plants, and the Y-axis indicates the gene clusters (that is, modules indicated by different colors) with the same expression pattern. The number in each module indicates the Pearson's correlation coefficient with the phenotype (from blue -1.00 to red 1.00) and in parentheses indicates the *P*-value of the correlation coefficient. The red boxes represent the module where *ARF2.1* is located; The yellow boxes represent the module where *ARF2.2* and *ARF3.1* are located; The green boxes represent the module where *ARF3.2* and *ARF4* are located. a, length from apex to node 10; b-h, length of IN4 (b) to IN10 (h); i, length of fiber cells; j, number of fiber cells, k-q, diameter of IN4 (k) to IN10 (q); r, base diameter; s, total leaf area of IN5 to IN10; t, total leaf circumference of IN5 to IN10; u, fresh weight of roots; v, dry weight of roots.

Supplementary Table 1 List of primer sequences used in this study

Primer	Sequence (5' -- 3')
**For gene cloning and vector construction**	
miR390b-3' RACE-Outer	GTTTAGGTCCCTTTGAAGCT
miR390b-3' RACE-Inner	GAGTTTCATTGCTTCTTCTTGCT
miR390b-5' RACE-Outer	GAAACTCAGGATGGATAGCGCCACT
miR390b-5' RACE-Inner	TCAAAAACCACATAAAAAGAGCC
miR390b-Full-F	ACCATTACTAGCTAGAGCATCG
miR390b-Full-R	TAGAGCAAACGATTAACTCACTCC
pro-miR390b-F	ATGAAACGAACCACTACTTCCGAG
pro-miR390b-R	CAAAGGGACCTAAACCGATGC
pro-miR390b-F-attB1	GGGGACAAGTTTGTACAAAAAAGCAGGCTATGAAACGAACCACTACTTCC GAG
pro-miR390b-R-attB2	GGGGACCACTTTGTACAAGAAAGCTGGGTCAAAGGGACCTAAACCGATGC
STTM-miR390	AAAAAGCAGGCTTCGGCGCTATCCCCTATCCTGAGCTTGTTGTTGTTGTTAT GGTCTAATTTAAATATGGTCTAAAGAAGAAGAATGGCGCTATCCCCTATCCT GAGCTTCACCCAGCTTTCT
**For positive identification of transgenic lines**	
HygII-F	CGGTCGGCATCTACTCTATTC
Hyg II-R	GAGGTCGCCAACATCTTCTT
Kan-F	CGGCGATACCGTAAAGCA
Kan-R	CGACCACCAAGCGAAACA
GUS-F	GTCGCGCAAGACTGTAACCA
GUS-R	CGGCGAAATTCCATACCTG
**For RT-qPCR**	
miR390-RT	AAGCTCAGGAGGGATAGCGCC
5.8S rRNA	GTCTGCCTGGGTGTCACGCAA
pri-miR390b-RT-F	AAATAGCTGCAAGGCTTGATGC
pri-miR390b-RT-R	AAAAACCACATAAAAAGAGCCCAT
TAS3.1-RT-F	CTCCTTCCTTGTCTATCCCTCCTG
TAS3.1-RT-R	CAAACTTGTTGATTTCTTCCTCCA
TAS3.2-RT-F	TCTTTCCCCATTTTCATCCAACTTA
TAS3.2-RT-R	GTGAGCCTCGCATGGTAAACAAG
ARF3.1-RT-F	CGAAGAGCAGCACAAGTTAAAT
ARF3.1-RT-R	CCTTGGATTGTAGTAAATGCGG
ARF3.2-RT-F	CCCGTGCTGAAAATTATCGAAT
ARF3.2-RT-R	TCTGTTGCATAGTCAAAGGAGT
PtActin-F	TCATCGGAATGGAAGCTGCTGGTA
PtActin-R	TAGTGGAACCACCACTGAGCACAA
**For tobacco transient co-expression**	
TAS3.1-F	***TCGAG***TCTTGTCTATCCCTCCTGAGCTGT***T***
TAS3.1-R	***CTAGA***ACAGCTCAGGAGGGATAGACAAGA***C***
TAS3.2-F	***TCGAG***TCTTATCTATCCCTCCTGAGCTAT***T***
TAS3.2-R	***CTAGA***ATAGCTCAGGAGGGATAGATAAGA***C***
tas3-F	***TCGAG***GATGTTGGAACGGCTCAGTCA***T***
tas3-R	***CTAGA***TGACTGAGCCGTTCCAACATC***C***
pMS4v2-35S	CACTGACGTAAGGGATGACGCA

Note: RT, reverse transcription.

Supplementary Table 2 Statistics of sRNA-Seq generated from 21 *Populus deltoides* I-69 × I-63 libraries

Sample	Raw reads	Clean reads	18-30 nt reads	Mapping to the genome	Matching known miRNAs	Unknown sRNAs
AP_T1	11,155,841	10,910,953	8,120,725	4,677,169	328,990	3,521,782
IN2_T1	12,007,209	11,778,538	8,853,020	6,405,603	136,784	2,452,159
IN3_T1	12,286,940	12,025,742	8,680,098	5,946,763	197,338	2,685,508
IN4_T1	13,089,871	12,803,239	9,102,907	6,348,911	237,716	2,784,735
IN5_T1	10,435,437	10,193,558	7,221,959	4,422,196	304,853	2,811,418
IN9_T1	11,398,543	11,123,282	7,292,983	4,139,088	691,015	3,213,316
LF_T1	9,420,459	9,207,679	6,589,997	4,152,501	225,995	2,534,117
AP_T2	9,475,446	9,242,571	6,833,178	4,108,781	208,466	2,754,726
IN2_T2	11,051,368	10,745,039	8,094,370	5,395,370	186,780	2,708,884
IN3_T2	13,318,415	13,121,893	10,044,107	6,094,104	464,445	4,117,141
IN4_T2	11,108,572	10,921,308	8,052,032	4,934,409	355,081	3,345,719
IN5_T2	9,864,659	9,687,540	6,751,388	4,444,555	225,963	2,349,957
IN9_T2	10,313,521	10,149,593	7,554,906	4,452,110	470,501	3,348,854
LF_T2	11,767,217	11,553,749	8,544,735	6,292,000	141,670	2,516,552
AP_T3	9,479,199	9,366,143	7,301,391	4,648,592	151,507	2,669,692
IN2_T3	10,065,919	9,813,950	6,398,669	4,432,425	100,245	1,964,446
IN3_T3	10,573,506	10,079,455	5,766,174	4,091,836	70,957	1,687,888
IN4_T3	10,756,648	10,540,930	7,687,473	5,003,247	219,048	2,831,159
IN5_T3	11,930,320	11,682,698	8,371,841	5,663,735	180,134	2,789,224
IN9_T3	11,993,315	11,790,710	8,535,924	5,679,774	240,799	2,925,622
LF_T3	13,217,457	12,893,635	8,464,411	6,046,771	130,941	2,628,154

Supplementary Table 3 Summary of RNA-Seq data from three OE-miR390b lines and WT

Sample	Raw reads	Clean reads	Aligned reads	Mapping rate
WT-1	44,325,192	40,943,862	30,687,424	74.95%
WT-2	51,013,864	42,077,636	30,792,414	73.18%
WT-3	44,196,222	42,605,498	31,775,180	74.58%
OE-42-1	47,910,948	46,230,894	34,922,818	75.54%
OE-42-2	55,717,958	53,571,848	41,459,254	77.39%
OE-42-3	50,656,036	49,034,186	37,378,760	76.23%
OE-44-1	48,620,688	45,341,894	35,112,762	77.44%
OE-44-2	53,801,118	51,748,362	39,815,190	76.94%
OE-44-3	60,815,552	58,433,146	45,203,882	77.36%
OE-65-1	47,343,266	45,334,610	34,395,368	75.87%
OE-65-2	51,840,968	50,214,652	38,328,844	76.33%
OE-65-3	53,188,528	51,170,038	39,365,110	76.93%

Supplementary Table 4 Common differentially expressed genes of three OE-miR390b lines

Up/down-regulation	Gene ID	Best-hit AT gene	AT gene description
up	Potri.001G120500	AT4G22590	Haloacid dehalogenase-like hydrolase (HAD) superfamily protein
up	Potri.002G037100	AT4G28530	NAC domain containing protein 74 (NAC074)
up	Potri.006G089000	AT3G09640	ascorbate peroxidase 2 (APX1B) (APX2)
up	Potri.018G069700	AT4G26260	myo-inositol oxygenase 4 (MIOX4) (ATCAX3) (ATHCX1)
down	Potri.016G115500	AT3G51860	cation exchanger 3 (CAX1-LIKE) (CAX3)
down	Potri.014G029100	AT3G19270	cytochrome P450, family 707, subfamily A, polypeptide 4 (CYP707A4)
down	Potri.001G189900	AT3G16360	HPT phosphotransmitter 4 (AHP4)
down	Potri.009G044600	AT2G29730	UDP-glucosyl transferase 71D1 (UGT71D1)
down	Potri.008G101675	AT3G17185	TAS3.2
down	Potri.003G173100	AT1G67360	Rubber elongation factor protein (REF)
down	Potri.013G041300	AT5G43700	AUX/IAA transcriptional regulator family protein (ATAUX2-11) (IAA4)
down	Potri.003G216500	AT1G55230	Family of unknown function (DUF716)
down	Potri.011G025200	AT5G54770	thiazole biosynthetic enzyme, chloroplast (ARA6) (THI1) (THI4)
down	Potri.008G166500	AT1G08650	phosphoenolpyruvate carboxylase kinase 1 (ATPPCK1) (PPCK1)
down	Potri.T101100	AT4G15210	beta-amylase 5 (BAM5) (BMY1) (RAM1)
down	Potri.001G097600	AT1G64110	P-loop containing nucleoside triphosphate hydrolases superfamily protein
down	Potri.015G143932	AT5G52300	CAP160 protein (LTI65) (RD29B)
down	Potri.001G416500	AT1G11530	C-terminal cysteine residue is changed to a serine 1 (ATCXXS1) (CXXS1)
down	Potri.004G231500	AT5G49760	Leucine-rich repeat protein kinase family protein
down	Potri.019G074400	AT4G32480	Protein of unknown function (DUF506)
down	Potri.013G093266	NA	NA
down	Potri.012G005900	AT5G24470	pseudo-response regulator 5 (APRR5) (PRR5)
down	Potri.001G130800	NA	NA
down	Potri.013G062200	NA	NA
down	Potri.010G047000	NA	NA
down	Potri.015G081300	AT1G12940	nitrate transporter2.5 (ATNRT2.5) (NRT2.5)
down	Potri.016G046400	AT5G06760	Late Embryogenesis Abundant 4-5 (LEA4-5)
